# Neonatal blood biomarkers related to development of hydrocephalus

**DOI:** 10.1186/s12987-026-00852-z

**Published:** 2026-08-01

**Authors:** Nicolas Hernandez Norager, Man-Hung Eric Tang, Michael Christiansen, Tina N. Munch, Nis Borbye-Lorenzen

**Affiliations:** 1 Department of Neurosurgery, Rigshospitalet, Copenhagen, Denmark; 2 Department of Sequencing and Bioinformatics, Statens Serum Institut, Copenhagen, Denmark; 3 Danish Center for Neonatal Screening, Department for Congenital Disorders, Statens Serum Institut, Copenhagen, Denmark; 4 Department of Biomedical Sciences, University of Copenhagen, Copenhagen, Denmark; 5 Department of Epidemiology, School of Public Health, University of Iowa, Iowa City, IA US; 6 Department of Clinical Medicine, University of Copenhagen, Copenhagen, Denmark

**Keywords:** Hydrocephalus, Dried blood spots, Prematurity, Newborn, Inflammation, Neurotrophic factors

## Abstract

**Background:**

Infantile hydrocephalus is a challenging condition with variable timing of clinical recognition. Early identification of newborns at increased risk could enable closer surveillance and earlier intervention. Neonatal blood samples collected routinely for newborn screening allow us to test whether specific blood biomarkers are associated to hydrocephalus.

**Methods:**

We used the data from an established nationwide Danish cohort study of 14,866 infants born from March 2009 to March 2011, with blood samples collected 2–8 days after birth and linked to longitudinal health registry data. The data included ten protein biomarkers spanning from inflammatory, cellular stress, growth, and neurotrophic pathways. Associations between neonatal biomarker concentrations and hydrocephalus were investigated using multivariable regression models adjusting for gestational age, age at sampling, and neonatal anthropometrics.

**Results:**

Among 14,866 infants, 41 were diagnosed with hydrocephalus (approximately 3 per 1,000 births). The primary biomarker analyses included 30 hydrocephalus cases diagnosed no later than the age of 2 years and 14,577 controls. Neonatal heat-shock protein 70 (HSP70) concentrations were significantly higher in infants who developed hydrocephalus. No significant differences were found for the remaining biomarkers, and subgroup analyses related to intraventricular hemorrhage did not show distinct biomarker profiles.

**Conclusions:**

In this nationwide cohort, elevated HSP70 in neonatal blood samples was associated with development of infantile hydrocephalus. No other of the inflammatory and neurotrophic biomarkers we investigated were significantly associated with hydrocephalus. These findings support further mechanistic investigation of HSP70 as a potential component of multifactorial early-life risk models of hydrocephalus.

**Supplementary Information:**

The online version contains supplementary material available at 10.1186/s12987-026-00852-z.

## Background

Infantile hydrocephalus is a clinically demanding condition and relatively frequent within the realm of congenital neurosurgical and pediatric practice [1, 2]. It is characterized by an abnormal amount of cerebrospinal fluid (CSF), resulting in dilation of the ventricular system, often with increased intracranial pressure, and a range of neurological symptoms [3]. The underlying causes are heterogeneous, ranging from intraventricular hemorrhage (IVH) and meningitis-related to structural abnormalities, genetic disorders, and developmental disturbances of CSF circulation, brain water homeostasis and CSF resorption [3–5]. Across etiologies, untreated infantile hydrocephalus is associated with substantial neurological morbidity and mortality, making early detection of the disease and identification of high-risk infants essential [6, 7].

In current practice, infants at high risk, especially those born extremely preterm or with IVH, are monitored using cranial ultrasound and serial clinical assessments (e.g. head circumference and neurological examination) [8, 9]. However, this approach detects hydrocephalus once ventricular dilation has already developed. Although some forms of infantile hydrocephalus, particularly structurally congenital cases, may be identified prenatally or neonatally, clinical recognition in other cases may occur later in infancy or childhood depending on etiology and severity. Earlier and more broadly applicable indicators of hydrocephalus risk, for example blood-based biomarkers, could therefore be of high value.

Most biomarker studies on infantile hydrocephalus have focused on CSF [10]. CSF studies report elevation in some inflammatory and stress-related biomarkers across underlying etiologies [11, 12]. These findings have advanced our understanding of the underlying pathophysiology and strengthened the evidence that inflammation contributes to the development of hydrocephalus [13]. However, this has limited utility for early risk estimation, since the invasiveness of CSF sampling does not allow it to be used as a screening tool.

Blood-based biomarkers are therefore an attractive alternative, if they can be robustly linked to the development of hydrocephalus. Blood tests in the neonatal period are already implemented in routine screening programs for other conditions, and the same samples could potentially contribute to early risk stratification for hydrocephalus [14]. Existing neonatal blood biomarker studies have mainly focused on infants who already have IVH or hydrocephalus, and it remains unclear whether early blood biomarker profiles can identify infants at increased risk of developing hydrocephalus [10, 15].

It is unlikely that any single blood-based biomarker will be clinically useful in isolation but could be in combination with pre- and perinatal clinical characteristics, cranial ultrasound and known risk factors to allow for earlier risk assessment. In the longer term, a multimodal approach could facilitate earlier - and potentially preventive - interventions before clinical hydrocephalus develops. In this study, we investigated whether 10 biomarkers in neonatal blood covering inflammation, cellular stress, growth and neurotrophic pathways are associated with hydrocephalus using data from an established nationwide Danish cohort of 14,866 newborns.

## Methods

### Study design and data sources

This population-based cohort study is based on the CODIBINE cohort, a Danish register-based birth cohort established to investigate associations between neonatal blood biomarkers and later health outcomes with a particular focus on prematurity-related outcomes. CODIBINE includes all preterm-born children (gestational age (GA) < 37 weeks) in Denmark between March 2009 and March 2011 and term-born controls matched on the date of birth and the hospital. Neonatal dried blood spot samples collected by heel prick as part of the national newborn screening program were analyzed for 10 biomarkers, spanning inflammatory, cellular stress, growth, and neurotrophic pathways. These biomarkers were selected when the CODIBINE cohort was established.

These biomarker data were linked to two national health registers by the personal civil registration number assigned to all residents of Denmark, which enables identification of included infants across biomarker data and national registers. Data on hospital contacts and diagnoses were retrieved from the Danish National Patient Register (DNPR), which records all ICD-coded hospital encounters nationwide [16]. Information on pregnancy, delivery, and perinatal characteristics was obtained from the Danish Medical Birth Register (MBR) [17]. Data access was delivered through the supercomputer ‘Forskermaskinen’ by the Danish Health Data Agency.

### Study population

The CODIBINE cohort comprises 7,936 preterm-born children and 7,936 term-born controls. Due to insufficient data from the DNPR (*n* = 756) and/or missing data in the MBR or unavailable biomarker measurements (*n* = 270), the included population consisted of 14,866 children (7,256 preterm and 7,610 term-born).

Children were selected based on the following ICD-10 diagnosis codes for any subtype of hydrocephalus: Q03*, Q046, Q046A, Q046B, Q046C, Q050-Q054, Q070, G91* and G942 recorded in the DNPR, where * indicates that all subdiagnoses for these codes are included. In addition, we required that a diagnosis was recorded on two separate dates to increase validity of the diagnosis. For each infant with a hydrocephalus diagnosis, all additional ICD-10 codes were reviewed to determine the presumed underlying cause. When more than one potential etiology preceded hydrocephalus, a single primary cause was assigned by a specialist in neurosurgery. For example, if both concussion and spontaneous intracranial hemorrhage were recorded prior to hydrocephalus, intracranial hemorrhage was classified as the primary cause, as it is more plausibly linked to subsequent hydrocephalus than a concussion. If no ICD-10 code indicated an underlying cause of hydrocephalus, the case was classified as “idiopathic infantile”. Furthermore, specifically ICD-10 codes for IVH (P52 and I61.5) were identified to allow for subgroup analyses. Afterwards, children with hydrocephalus diagnosed after 2 years of age were excluded from the primary analyses, as the underlying etiology was not likely to have been more than minimally present at the time of neonatal blood sampling.

To allow comparison with other inflammatory conditions not related to the central nervous system (CNS), we defined a control group with non-CNS inflammatory conditions, including children with one of the following ICD-10 codes within the first 92 days after birth: A40, A41, P22.0, P23*, P24*, P29.0, P29.3, P29.4, P29.8–P29.9, P36*, P37.0–P37.4, P37.8–P37.9, P39.2, P77* (* indicates that all sub-diagnoses for these codes are included).

### Neonatal blood sampling and biomarker measurements

The dried blood spot samples analyzed in CODIBINE were retrieved from the Danish Neonatal Screening Biobank that stores leftover samples from the Danish newborn screening program. Blood was obtained by heel prick after birth, and dried blood spot samples were analyzed for 10 biomarkers using a multiplex immunoassay as described in detail previously [18, 19]. Only samples obtained on day 2–8 were included in the data analyses, 252 individuals with blood collected 9 days or later after birth were removed.

The biomarker panel comprised the following inflammatory, stress-related, growth, and neurotrophic markers to cover a broad range of markers relevant for assessing the outcomes of being born preterm in relation to organ, brain and immune system maturation: heat-shock protein 70 (HSP70), interleukin-18 (IL-18), glia cell protein S100B (S100B), monocyte chemoattractant protein-1 (MCP-1), C-reactive protein (CRP), soluble tumor necrosis factor receptor I (sTNF-RI), epidermal growth factor (EGF), vascular endothelial growth factor A (VEGF-A), brain-derived neurotrophic factor (BDNF) and neurotrophin-3 (NT-3). The panel was not selected specifically for hydrocephalus but was used here as a predefined exploratory neonatal dried blood spot panel covering biological domains relevant to prematurity, inflammation, brain injury, and neurodevelopment.

Size for gestational age variables were calculated based on the sex-specific intrauterine growth curves as described in Maršál et al. [20]. Small and large-for-gestational-age were defined as being +/- 2SD deviations from the estimated curve.

We calculated the following four development metrics using birth measures as described in Streja et al. [21]: the cephalization index defined as the head circumference / weight, the head/abdominal circumference ratio, the ponderal index defined as the head circumference / length^3^ and the birth placenta weight ratio defined as the weight at birth over the placenta weight.

### Statistical analysis

Wilcoxon rank-sum tests were performed to compare the log-transformed distributions of each biomarker measured in blood collected from children diagnosed with hydrocephalus and controls without hydrocephalus. Bonferroni multiple-testing correction was performed over the 10 tests to mitigate false discoveries due to assay intrinsic variability. Anthropometrics such as weight, length, head and abdominal circumference, and developmental indices were also compared using an analysis of covariance (ANCOVA) adjusted for gestational weeks. Two-sided Fisher’s exact tests were performed to compare the proportions of different GA groups between subjects with hydrocephalus vs. all included subjects without hydrocephalus.

Log-transformed biomarker concentrations were modeled by linear regression as the dependent variables and the binary variable hydrocephalus with age cutoffs at diagnosis under 2 years and 1 year was included as the primary independent variable in the two analyses, together with the covariables age at blood collection (days), GA (weeks) and the anthropometrics weight, length, head circumference and abdominal circumference.

Bootstrapped linear regression was performed by running the linear regression model described above for log-transformed HSP70 as dependent variable on the independent variable hydrocephalus diagnosed under the age of 2. The bootstrap was performed one thousand times on random samples of 80% of the data with replacement. The mean, standard error and 95% confidence interval of the regression coefficient obtained for the variable hydrocephalus diagnosed under the age of 2 years was calculated. All statistical analyses were performed using R 4.4.3 and visualizations using ggplot2 3.5.2 and ggpubr 0.6.1.

In accordance with Danish data protection and disclosure control requirements, we do not report exact cell counts for any category with fewer than five individuals. Such values are presented as ‘<5’ (or suppressed), and categories were collapsed where necessary to ensure a minimum cell size of five before reporting results.

## Results

### Study population and characteristics

Among the 14,866 children included in this study, we identified 41 children with an ICD code of hydrocephalus. The incidence of hydrocephalus in the cohort was thus approximately 3 per 1,000 births. Of these, 33 were born preterm (< 37 GA), corresponding to a preterm incidence of 4.5 per 1,000 live births, and 8 were born at term, corresponding to a term incidence of 1 per 1,000 births.

Underlying etiologies of hydrocephalus are summarized in Table 1. Of the 41 hydrocephalus cases, 13 (32%) were hemorrhage-related (predominantly IVH) and 17 (41%) had no identifiable cause and were classified as idiopathic hydrocephalus. The remaining 11 cases (27%) were attributed to structural malformations, tumors, trauma or meningitis.

**Table 1:** Etiologies of hydrocephalus

Etiologies of hydrocephalus	*n* (%)	ICD10-codes*
Hemorrhage-related	13 (32)	I615, I620, I63, P521, P522
Idiopathic	17 (41)	
Structural malformations, tumors, trauma or meningitis	11 (27)	C71, G008, Q030, Q052, Q070, S060

*I61.5, intracerebral hemorrhage, intraventricular; I62.0, subdural heemorrhage (acute/nontraumatic); I63, cerebral infarction; P52.1, intraventricular (nontraumatic) hemorrhage of fetus and newborn; P52.2, other intracranial (nontraumatic) hemorrhage of fetus and newborn; C71, malignant neoplasm of brain; G00.8, other bacterial meningitis; Q03.0, congenital hydrocephalus; Q05.2, spina bifida with hydrocephalus; Q07.0, Arnold–Chiari syndrome; S06.0, concussion

The clinical characteristics are summarized in Table 2. Preterm hydrocephalus predominantly affects male cases (70%) and we found significantly lower mean GA (mean 31 vs. 34 weeks) and lower birth weight (mean 1,823 g SD 767 vs. 2,322 g SD 653 *p* < 0.001) and other anthropometric measurements such as length, head and abdominal circumference compared to preterm controls.

**Table 2:** Subjects’ characteristics

	Preterm		Full-term	
	Control	Hydrocephalus	Control	Hydrocephalus
	7223	33	7602	8
Weight (g)| mean (SD)| NA	2332 (653) | 19	1823 (767) | 0 ***	3548 (494) | 5	3161 (497) | 0 *
Placenta weight (g)| mean (SD)| NA	657 (266) | 252	618 (281) | <5	689 (166) | 149	630 (191) | <5
Length (cm)| mean (SD)| NA	46.5 (4.0) | 546	44.1 (4.8) | 7 ***	52.0 (2.3) | 56	51.4 (2.7) | 0
Head circumference (cm)| mean (SD)| NA	32.1 (2.6) | 894	30.3 (3.9) | 8 ***	35.0 (1.6) | 97	35.3 (4.3) | <5
Abdominal circumference (cm)| mean (SD)| NA	29.3 (3.2) | 1724	27.6 (3.9) | 17 **	33.3 (2.2) | 190	32.1 (2.2) | <5
Cephalization index | mean (SD)| NA	0.014 (0.004) | 894	0.017 (0.005) | 8 ***	0.010 (0.001) | 97	0.012 (0.002) | <5 ***
Head abdominal circumference ratio | mean (SD)| NA	1.110 (0.097) | 1770	1.128 (0.070) | 18	1.055 (0.060) | 196	1.103 (0.169) | <5 *
Ponderal index | mean (SD)| NA	0.023 (0.005) | 548	0.021 (0.003) | 7 *	0.025 (0.003) | 56	0.023 (0.002) | 0.
Birth placenta weight ratio | mean (SD)| NA	3.941 (1.396) | 267	3.291 (1.101) | <5 **	5.352 (1.157) | 151	5.526 (1.646) | <5
Size for gestational age (%)				
SGA	763 (11)	0(0.0)	127 (2)	0(0.0)
LGA	353 (5)	0(0.0)	335 (4)	0(0.0)
AGA	6088 (84)	32 (100.0) *	7135 (94)	7(100.0)
NA		1		1
Gestational age (weeks) | mean (SD)	34.0 (2.5)	31.3 (4.1) ***	39.6 (1.3)	39.0 (1.3)
Gestational age (%)				
28 and less	353 (5)	11 (48) *		
29	182 (3)	#		
30	231 (3)			
31	251 (4)			
32	417 (6)			
33	596 (8)			
34	1031 (14)			
35	1516 (21)	5 (22)		
36	2646 (37)	7 (30)		
37			503 (7)	#
38			1164 (15)	
39			1735 (23)	
40			2227 (29)	
41			1522 (20)	
42 or more			451 (6)	
NA		10		
Gender (%)				
Female	3368 (47)	10 (30)	3527 (46)	#
Male	3855 (53)	23 (70)	4075 (54)	

The characteristics of the study subjects, stratified by gestational term (preterm vs. full-term) and hydrocephalus (HC) as per the Danish National Patient Registry. Anthropometric measures between HC and non-HC were compared using an analysis of covariance adjusted for gestational weeks. Proportions of categorical variables were compared using a Fisher’s exact test. Significant differences are indicated by symbols (*** *p* ≤ 0.001; ** 0.001 < *p* ≤ 0.01; * 0.01 < *p* ≤ 0.05;. 0.05 < *p* < 0.1). # indicates that there is too little data for these characteristics to allow division in GA weeks and gender as per data regulation rules. SGA, small for gestational age (GA), AGA, average for GA, LGA, large for GA, NA, not available

In addition, preterm hydrocephalus shows higher values of cephalization index (mean 0.017 vs. 0.014 *p* < 0.001), indicative of large head size relative to body weight, and lower ponderal index.

Low birth weight is a marker of extreme prematurity and intrauterine growth restriction [22] and the low values in length, head and abdominal circumferences are consistent with this observation.

For the biomarker analyses, 11 children were excluded either because the heel-prick blood sample was collected late or because their first hydrocephalus diagnosis was registered after 2 years of age, leaving 30 children with hydrocephalus (25 preterm and 5 term). Of these cases 10 (33%) were hemorrhage-related, 15 (50%) were idiopathic and the remaining 5 (17%) were either structural malformations, tumors, trauma or meningitis. After exclusions for missing data, sampling ≥ 9 days after birth, or missing biomarker measurements, 14,577 controls remained.

### Biomarker profiles related to development of hydrocephalus

**Fig. 1:**
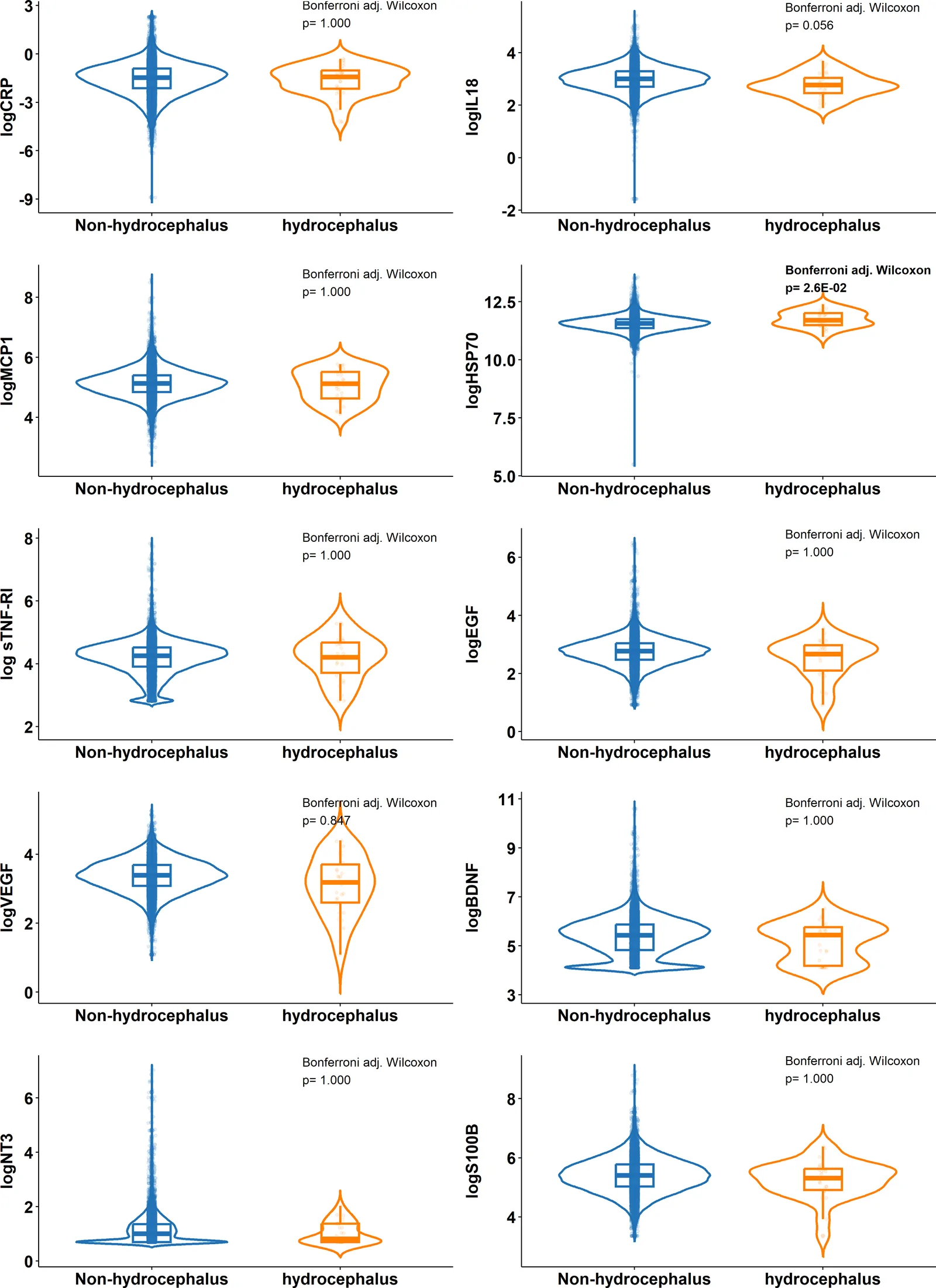
Biomarker levels in hydrocephalus and non-hydrocephalus individuals. Log-transformed distributions of the blood biomarkers in individuals diagnosed with hydrocephalus (*n* = 30) before the age of 2 years and those without hydrocephalus (*n* = 14,577) are shown. Non-parametric Wilcoxon rank-sum tests were used for group comparisons and Bonferroni-correction for multiple testing was applied

In our primary analyses comparing children with and without hydrocephalus before 2 years of age, most neonatal biomarkers measured in blood collected 2–8 days after birth (*n* = 30) showed no significant differences in the non-parametric tests (see Fig. 1 and Supplementary Table 1). However, neonatal HSP70 concentrations were higher in children who developed hydrocephalus than in those who did not (Bonferroni-adjusted *p* = 0.026). This association remained statistically significant when running a linear regression model on the dependent variable log-transformed HSP70 concentration against the independent variable hydrocephalus adjusted for the confounders GA, age at blood sampling, and neonatal anthropometrics (1.289 fold change, 95% CI [1.095–1.519], *p* = 0.002, Supplementary Table 2). A bootstrapped linear regression on the log-transformed HSP70 concentration using the same model was performed one thousand times on random samples of 80% of the data with replacement. The bootstrapped mean effect, standard error and 95% confidence intervals for the variable hydrocephalus diagnosed under the age of 2 years matched the ones calculated in the regression analysis (mean fold change 1.283, 95%CI [1.080–1.557] vs. 1.289, 95%CI [1.095–1.519]).

IL-18 showed marginally lower levels in infants with hydrocephalus (Bonferroni-adjusted Wilcoxon rank-sum test *p* = 0.056) which was confirmed to be non-significant when performing a linear regression after adjustment for the above-mentioned confounders (0.867 fold change, 95% CI [0.687–1.094], *p* = 0.230, Supplementary Table 3). The remaining biomarkers (S100B, MCP-1, CRP, sTNF-RI, EGF, VEGF-A, BDNF and NT-3) showed no significant differences.

Similarly, in an analysis restricted to preterm infants (*n* = 25), the HSP70 levels were again higher for hydrocephalus cases in the non-parametric test (*p* = 0.028), and the association remained significant in the linear regression model adjusted for the same covariables (1.361 fold change, 95% CI [1.114–1.662], *p* = 0.003, Supplementary Table 4). IL-18 levels were not significantly different in hydrocephalus cases in this subgroup (Bonferroni-adjusted *p* = 0.454). A similar analysis among term-born infants (*n* = 5) was not performed, due to insufficient sample size.

To further understand our findings on the biomarker HSP70, we defined a non-CNS inflammatory control group, which includes other inflammatory conditions not related to the CNS (Material and Methods). We first performed a linear regression of the log-transformed HSP70 dependent variable on the independent variable non-CNS inflammatory group, adjusted by the same covariables, using the population without hydrocephalus or any disease defined in the non-CNS inflammation group as controls. We found that HSP70 was elevated in children with a non-CNS inflammation diagnosis (fold change 1.031, 95% CI [1.007–1.055], *p* = 0.010, Supplementary Table 5), however the effect was markedly smaller. When comparing hydrocephalus with the control group with non-CNS-related inflammatory conditions, we found that HSP70 was more elevated in the hydrocephalus group (1.273 fold change, 95% CI [1.060–1.529], *p* = 0.010, Supplementary Table 6). The difference in effect was similar to that observed when comparing hydrocephalus cases with all controls (1.273 vs. 1.289 fold change).

### Subgroup analyses according to age at diagnosis

Our primary analysis was performed by comparing children with and without hydrocephalus with a 2-year age cut off, which was chosen as a pragmatic balance between biological plausibility and preservation of sufficient sample size in a rare disease. To elucidate whether a more stringent cut-off would allow us to find more biomarker associations, we repeated the analysis using a cut off at one year of age which reduced our number of cases by 5. The non-parametric tests confirmed our previous finding for HSP70 (Bonferroni-adjusted *p* = 0.036, Supplementary Fig. 1, Supplementary Table 7) and the linear regression analysis showed a similar effect size for hydrocephalus diagnoses under the age of 1 (1.341 fold change, 95%CI [1.124–1.601], *p* = 0.001, Supplementary Table 8).

### IVH-related subgroup analyses

We compared biomarker profiles between hydrocephalus cases classified as IVH-related hydrocephalus (*n* = 10) and non-IVH-related hydrocephalus (*n* = 20) (Supplementary Fig. 2 and Supplementary Table 9). No biomarker differed significantly between groups. However, these analyses may have been underpowered to detect modest effects. In subgroup analyses restricted to infants with IVH, we compared biomarker levels in those who subsequently developed hydrocephalus (*n* = 10) versus those who did not (*n* = 105) (Supplementary Fig. 3 and Supplementary Table 10). Again, no biomarker was statistically significant, and no clear patterns emerged. To clarify if the cases of hydrocephalus not caused by IVH by themselves had elevated HSP70, we compared non-IVH-related cases (*n* = 20) with controls (Supplementary Fig. 4 and Supplementary Table 11). No biomarker differed significantly, but the analysis may have lacked sufficient sample size.

## Discussion

In this nationwide, register-based birth cohort with neonatal dried blood spots linked to diagnosis and clinical characteristics at birth, we examined whether a panel of 10 neonatal blood biomarkers representing inflammatory, cellular stress, growth and neurotrophic pathways are associated with development of infantile hydrocephalus. HSP70 emerged as the only biomarker with a significant association. Higher neonatal HSP70 concentrations were related to hydrocephalus, both in the full cohort and in analyses restricted to preterm infants and we were able to replicate the association by bootstrapping. Our further investigations on HSP70 suggested that elevated levels of this marker could be part of a broader stress-response since levels were also elevated in a non-CNS inflammation control group, but the levels were even higher in the hydrocephalus group when comparing both groups.

For the remaining biomarkers (IL-18, MCP-1, CRP, sTNF-RI, EGF, VEGF-A, BDNF, NT-3 and S100B) no statistically significant associations were found. We also examined whether biomarker profiles varied by etiology and within newborns with IVH. In these subgroup analyses, no biomarkers were found to be statistically significant.

### Epidemiological findings

In the cohort, we identified 41 children with hydrocephalus, corresponding to an incidence of approximately 3 per 1,000 births, with most cases occurring in the preterm group of infants (81%). Idiopathic hydrocephalus accounted for roughly 40%, IVH-related hydrocephalus for about one third, with the remainder attributable to structural malformations, infection and trauma. This distribution is in line with previous literature in which IVH is the most frequent acquired cause and a substantial part of cases remains without an identified cause. In a large cohort of 411 infants, for example, about 40% had a documented extrinsic cause - most commonly IVH - while the remaining approximately 60% were classified as idiopathic hydrocephalus [5].

However, our cohort is not directly comparable to published population-based data, as it is deliberately enriched for preterm infants. Approximately half of the children are preterm, compared with 7–8% expected in a European population [23]. This naturally inflates the hydrocephalus incidence and shifts the etiology towards IVH-related and other prematurity-associated causes. When analyzed separately, we observed a preterm incidence of 4.5 per 1,000 and a term born incidence of 1 per 1,000 births. These fall within the range reported in previous studies, which describe congenital hydrocephalus incidences of roughly 0.3–2.5 per 1,000 births overall and just under 1 per 1,000 in high-income countries [1, 24, 25]. The male predominance among hydrocephalus cases and the lower birth weight are likewise in line with prior epidemiological studies [24, 25]. We find that preterm hydrocephalus cases have a higher cephalization and lower ponderal indexes when compared to preterm controls. Together with the low anthropometrics, this could be indicative of growth restriction. These values should be interpreted with caution, though, due to the high fraction of missing values in the anthropometric variables (NAs in Table 2) and additional studies in larger cohorts may be needed to confirm these findings.

Overall, the epidemiological profile of our cohort is broadly consistent with previous reports, which supports that our inclusion strategy in fact captured most hydrocephalus cases in the study period.

### HSP70 as a blood biomarker related to hydrocephalus

Our main finding was higher neonatal HSP70 concentrations in neonates who developed hydrocephalus. Other groups have reported higher levels of blood HSP70 in neonates with perinatal asphyxia/hypoxic–ischemic encephalopathy, children with febrile and afebrile seizures, and in pediatric acute brain injury [26–28].

HSP70 is a stress-response protein and has been linked to neuroprotective effects [29–31]. There are few studies of HSP70 in the human neonatal brain, but in a neonatal mouse model of hypoxic–ischemic brain injury, transgenic overexpression of HSP70 has been associated with significantly reduced lesion size, supporting a potential neuroprotective role of HSP70 in the context of perinatal hypoxia–ischemia [31]. Thus, within the framework of the current evidence on HSP70, the higher neonatal HSP70 we observe in infants who later develop hydrocephalus most likely reflects an early, non-specific response to the underlying CNS insult (e.g. IVH/brain stress leading to hydrocephalus) – rather than hydrocephalus itself [12, 13].

It is important to note that we did not observe a consistent elevation in the other circulating inflammatory markers included in our panel. This observation supports our interpretation that the increased levels of neonatal HSP70 likely reflect brain stress or injury burden in newborns, rather than indicating systemic inflammation. Given the timing of sampling (2–8 days after birth), we cannot exclude that some infants already had hydrocephalus at the time of blood sampling, and our data should therefore not be overinterpreted as evidence for a purely pre-symptomatic biomarker.

Clinically, these characteristics imply that HSP70 is unlikely to serve as a standalone diagnostic marker for hydrocephalus. However, because we measured HSP70 in blood in all neonates at an early time point, our study differs fundamentally from most existing hydrocephalus biomarker work, which predominantly investigates CSF after hydrocephalus has developed, often at the time of neurosurgical intervention [11, 12, 26, 28]. Our findings indicate that a blood biomarker measured shortly after birth is associated with the development of hydrocephalus. In practice, the overlap in HSP70 concentrations between infants who do and do not develop hydrocephalus, and the wide range of conditions capable of raising HSP70, suggest that, if confirmed in larger studies and mechanistic work, HSP70 will be most useful as one component of a multifactorial risk profile rather than as an isolated screening test.

### IL-18 in neonates and its relation to prematurity

IL-18 has previously been implicated in the pathophysiology of hydrocephalus in the neonatal period or early infancy in CSF-based studies, where markedly elevated CSF IL-18 concentrations and associations with white matter damage have been reported [11, 12, 32]. In our cohort, neonatal blood IL-18 levels were not significantly different between hydrocephalus cases and controls, apart from in the unadjusted analysis, where hydrocephalus diagnosis was restricted to occur within 1 year. This difference disappeared after adjustment for confounders, with the driving confounder being GA. Hydrocephalus cases were more likely to be preterm, and increasing prematurity was associated with progressively lower blood IL-18 levels, as described in detail in previous articles from our group [18, 33, 34]. Our findings therefore do not contradict the current literature but instead highlight the need to adjust carefully for prematurity when interpreting IL-18. Thus, GA must be explicitly modeled in neonatal IL-18 biomarker studies.

### Biomarker profiles across different etiologies

On the basis of CSF biomarker studies, we hypothesized that hydrocephalus secondary to IVH would show increased levels of inflammatory and stress markers compared to congenital or structural hydrocephalus. Several studies report markedly elevated CSF cytokines and chemokines in post-hemorrhagic and post-infectious hydrocephalus, whereas congenital obstructive hydrocephalus exhibits only modest or minimal cytokine elevation compared with controls [12, 13, 35]. However, in our cohort, neonatal blood biomarkers did not differ significantly between IVH-related and non-IVH-related hydrocephalus cases. Given the small subgroup sizes (*n* = 10 and *n* = 20), the analysis was only powered to detect quite large differences, thus our data do not provide evidence for different etiology-specific neonatal blood biomarker profiles for hydrocephalus. We also found no significant differences in biomarker levels in non-IVH-related hydrocephalus cases versus non-hydrocephalus controls, although this analysis was limited by the small number of cases (Supplementary Fig. 4 and Supplementary Table 11).

### Biomarkers in infants with IVH

We also explored whether blood biomarkers could distinguish between infants with IVH that did and did not subsequently develop hydrocephalus. None of our biomarkers showed significant changes in this sub analysis, most probably due to the small sample size. Our findings therefore do not provide evidence that the blood biomarkers examined can reliably predict progression from IVH to hydrocephalus. Our findings should be interpreted in the context of the literature. S100B, which is also included in our biomarker panel, is one of the most extensively studied blood biomarkers of neonatal brain injury. Elevated concentrations in maternal blood, cord blood, and early neonatal blood or urine are consistently associated with severe IVH and white-matter damage in preterm infants and are generally interpreted as reflecting the magnitude of early astroglia injury [10, 36–38]. Importantly, most of these studies compare infants with severe IVH or brain injury to infants without IVH or with milder grades, whereas our analysis was conducted among infants who all had IVH and contrasts those who did versus did not progress to hydrocephalus. In our cohort, neonatal S100B did not show a robust independent association with this progression. Given the small number of hydrocephalus cases, more modest effects cannot be excluded, but our findings support the view that early blood biomarkers primarily index overall brain injury burden, whereas the specific evolution from IVH to hydrocephalus likely depends on additional factors not captured by these systemic markers.

### Limitations

Only 30 hydrocephalus cases were available for the primary biomarker analyses after the exclusion criteria, and several clinically relevant subgroups were too small to provide adequate statistical power to reliably detect significant differences. This limited case number reflects the rarity of hydrocephalus, even in a nationwide neonatal cohort, and was further reduced by the strict case definition, exclusion of late-diagnosed cases, and restriction to appropriately timed neonatal blood samples. The study therefore only had adequate power to detect relatively large biomarker differences, and more modest associations cannot be excluded. Because the analysis was limited to a predefined panel of ten biomarkers, the absence of additional significant associations cannot exclude inflammatory or other biological processes not captured by this panel. Hydrocephalus diagnoses and underlying causes were obtained from ICD-10 codes in national registries, which introduces the possibility of misclassification of both case status and etiology. This limitation is partly mitigated by the repeatedly validated completeness of the Danish National Patient Register and the generally high validity reported for diagnoses related to surgical specialties, including pediatric surgical diagnoses [39]. In addition, we did not have access to imaging data, detailed neurosurgical procedure information, or precise data on the timing of IVH in relation to blood sampling, limiting etiologic profiling. Dried blood spots were collected on postnatal days 2–8. Although most infants were likely presymptomatic with respect to clinically recognized hydrocephalus at that time, some may already have had ventriculomegaly or raised intracranial pressure, which could influence biomarker levels. Conversely, hydrocephalus may present during infancy even when the underlying predisposition or pathology is congenital or already present early in life. Furthermore, registry-based diagnosis may be delayed until hospital discharge or later clinical coding. Thus, the cohort may include infants at different stages of the disease process at the time of blood sampling, including infants with evolving but not yet clinically recognized hydrocephalus. Finally, biomarkers were measured at a single time point from one neonatal sample, which precluded assessment of temporal changes in biomarker levels and their duration. Temporal dynamic patterns, especially concerning IVH and evolving hydrocephalus, could provide additional prognostic information.

## Conclusion

In this nationwide cohort using neonatal dried blood spots, higher neonatal HSP70 concentrations were associated with hydrocephalus, both overall and among preterm infants. We did not identify blood biomarkers that discriminated IVH-related from non–IVH-related hydrocephalus or predicted progression to hydrocephalus among infants with IVH. Our data suggest that HSP70 could contribute to future multifactorial risk models for hydrocephalus, if confirmed in larger cohorts and mechanistic studies.

## Supplementary Information

Below is the link to the electronic supplementary material.

Supplementary Material 1

## Data Availability

The data in this study consist of registry-linked data and neonatal dried blood spot biomarker measurements. Due to Danish data protection legislation and the terms of the approvals, the raw data cannot be made publicly available. Access may be granted to researchers subject to permission from the relevant Danish authorities/data holders and completion of required approvals and data processing agreements.

## Abbreviations

ANCOVA: Analysis of covariance
BDNF: Brain-derived neurotrophic factor
CRP: C-reactive protein
CSF: cerebrospinal fluid
DNPR: Danish National Patient Register
EGF: Epidermal growth factor
GA: Gestational age
HSP70: Heat-shock protein 70
ICD-10: International Classification of Diseases, 10th Revision
IL-18: Interleukin-18
IVH: Intraventricular hemorrhage MBR: Danish Medical Birth Register
MCP-1: Monocyte chemoattractant protein-1
NT-3: Neurotrophin-3
S100B: Glia cell protein S100B
sTNF-RI: Soluble tumor necrosis factor receptor I
VEGF-A: Vascular endothelial growth factor A
