# Supplementary material for: Neonatal blood biomarkers related to development of hydrocephalus
Source: Fluids Barriers CNS. 2026 Aug 1;23:103. doi: 10.1186/s12987-026-00852-z (PMC13520248; doi:10.1186/s12987-026-00852-z)
Supplement: Supplementary file 1 [file 12987_2026_852_MOESM1_ESM.docx]

**Supplementary material**

Nicolas H. Norager *et al.* Neonatal Blood Biomarkers Related to Development of Hydrocephalus

**Supplementary figure 1: Biomarker levels in hydrocephalus diagnosed before the age of 1 year and non-hydrocephalus individuals**
Log-transformed distributions of the blood biomarkers in individuals diagnosed with hydrocephalus (n= 25) before the age of 1 year and those without hydrocephalus (n=14577) are shown. Non-parametric Wilcoxon rank-sum tests were used for group comparisons and Bonferroni-correction for multiple testing was applied.

**Supplementary figure 2: Biomarker levels in hydrocephalus individuals with and without intraventricular hemorrhage**
Log-transformed distributions of the blood biomarkers in individuals diagnosed with hydrocephalus before the age of 2 years without intraventricular hemorrhage (n= 20) and hydrocephalus with IVH (n=10) are shown. Non-parametric Wilcoxon rank-sum tests were used for group comparisons and Bonferroni-correction for multiple testing was applied.

**Supplementary figure 3: Biomarker levels in individuals with intraventricular hemorrhage without and with hydrocephalus**
 Log-transformed distributions of the blood biomarkers in individuals diagnosed with intraventricular hemorrhage without (n= 105) and with hydrocephalus diagnosed before the age of 2 years (n=10) are shown. Wilcoxon rank-sum tests were used for group comparisons and Bonferroni-correction for multiple testing was applied.

**Supplementary figure 4: Biomarker levels in individuals with hydrocephalus not caused by intraventricular hemorrhage and those without hydrocephalus** Log-transformed distributions of the blood biomarkers in individuals diagnosed with hydrocephalus not caused by intraventricular hemorrhage diagnosed before the age of 2 years (n= 20) and those without hydrocephalus (n=14472) are shown. Wilcoxon rank-sum tests were used for group comparisons and Bonferroni-correction for multiple testing was applied.

| **Biomarker** | **Wilcoxon p-value** | **Bonferroni adjusted p-value** |
| --- | --- | --- |
| **logCRP** | 0.689 | 1 |
| **logIL18** | 0.006 | 0.056 |
| **logMCP1** | 0.692 | 1 |
| **logHSP70** | 0.003 | 0.026 |
| **logSTNF_RI** | 0.732 | 1 |
| **logEGF** | 0.177 | 1 |
| **logVEGF** | 0.085 | 0.847 |
| **logBDNF** | 0.276 | 1 |
| **logNT3** | 0.379 | 1 |
| **logS100B** | 0.337 | 1 |

**Supplementary table 1: Unadjusted and adjusted p-values comparing biomarker levels in hydrocephalus and non-hydrocephalus individuals.** Individuals diagnosed with hydrocephalus (n= 30) before the age of 2 years and those without hydrocephalus (n=14577) were analyzed. Non-parametric Wilcoxon rank-sum tests were used for group comparisons and Bonferroni-correction for multiple testing was applied.

| HSP70 vs HC (preterm + full-term) | **Estimate** | **Std. Error** | **Lower 95%CI** | **Upper 95%CI** | **Fold change** | **FC lower 95%CI** | **FC upper 95%CI** | **z value** | **Pr(>\|z\|)** |
| --- | --- | --- | --- | --- | --- | --- | --- | --- | --- |
| **(Intercept)** | 11.878 | 0.083 | 11.714 | 12.041 |  |  |  | 142.315 | 0.00E+00 |
| **Hydrocephalus diagnosed under 2 yrs** | 0.254 | 0.084 | 0.090 | 0.418 | 1.289 | 1.095 | 1.519 | 3.041 | 2.36E-03 |
| **Age at collection (days)** | -0.008 | 0.004 | -0.015 | -0.001 | 0.992 | 0.985 | 0.999 | -2.120 | 3.40E-02 |
| **Birth weight** | -3.06E-05 | 1.10E-05 | -5.23E-05 | -8.98E-06 | 1.000 | 1.000 | 1.000 | -2.773 | 5.57E-03 |
| **Length** | -0.002 | 0.002 | -0.005 | 0.002 | 0.998 | 0.995 | 1.002 | -0.983 | 3.26E-01 |
| **Head circumference** | -0.008 | 0.002 | -0.011 | -0.004 | 0.993 | 0.989 | 0.996 | -3.889 | 1.01E-04 |
| **Abdominal circumference** | 0.007 | 0.002 | 0.003 | 0.010 | 1.007 | 1.003 | 1.010 | 3.911 | 9.24E-05 |
| **Gestational age (weeks)** | -0.002 | 0.002 | -0.006 | 0.001 | 0.998 | 0.994 | 1.001 | -1.300 | 1.94E-01 |

**Supplementary table 2: Coefficients of the logistic regression model for HSP70 against hydrocephalus adjusted for the confounders GA, age at blood sampling, and neonatal anthropometrics (birth weight, length, head circumference, abdominal circumference).**

| IL18 vs HC (preterm + full-term) | **Estimate** | **Std. Error** | **Lower 95%CI** | **Upper 95%CI** | **Fold change** | **FC lower 95% CI** | **FC 95% upper CI** | **z value** | **Pr(>\|z\|)** |
| --- | --- | --- | --- | --- | --- | --- | --- | --- | --- |
| **(Intercept)** | 2.110 | 0.119 | 1.877 | 2.342 |  |  |  | 17.797 | 5.30E-70 |
| **Hydrocephalus diagnosed under 2 yrs** | -0.142 | 0.119 | -0.375 | 0.090 | 0.867 | 0.687 | 1.094 | -1.200 | 0.230 |
| **Age at collection (days)** | -0.005 | 0.005 | -0.015 | 0.005 | 0.995 | 0.985 | 1.005 | -0.938 | 0.348 |
| **Birth weight** | 1.81E-05 | 1.57E-05 | -1.27E-05 | 4.88E-05 | 1.000 | 1.000 | 1.000 | 1.152 | 0.249 |
| **Length** | 0.001 | 0.002 | -0.003 | 0.006 | 1.001 | 0.997 | 1.006 | 0.588 | 0.556 |
| **Head circumference** | -0.009 | 0.003 | -0.014 | -0.004 | 0.991 | 0.986 | 0.996 | -3.266 | 0.001 |
| **Abdominal circumference** | 0.004 | 0.002 | -0.001 | 0.009 | 1.004 | 0.999 | 1.009 | 1.736 | 0.083 |
| **Gestational age (weeks)** | 0.026 | 0.002 | 0.021 | 0.030 | 1.026 | 1.021 | 1.031 | 10.473 | 1.46E-25 |

**Supplementary table 3: Coefficients of the logistic regression model for IL18 against hydrocephalus adjusted for the confounders GA, age at blood sampling, and neonatal anthropometrics (birth weight, length, head circumference, abdominal circumference).**

| HSP70 vs HC (preterm only) | **Estimate** | **Std. Error** | **Lower 95%CI** | **Upper 95%CI** | **Fold change** | **FC lower 95% CI** | **FC upper 95% CI** | **z value** | **Pr(>\|z\|)** |
| --- | --- | --- | --- | --- | --- | --- | --- | --- | --- |
| **(Intercept)** | 12.055 | 0.127 | 11.807 | 12.304 |  |  |  | 95.111 | 0.00E+00 |
| **Hydrocephalus diagnosed under 2 yrs** | 0.308 | 0.102 | 0.108 | 0.508 | 1.361 | 1.114 | 1.662 | 3.015 | 0.003 |
| **Age at collection (days)** | -0.004 | 0.005 | -0.015 | 0.006 | 0.996 | 0.985 | 1.006 | -0.815 | 0.415 |
| **Birth weight** | -1.99E-05 | 1.90E-05 | -5.71E-05 | 1.74E-05 | 1.000 | 1.000 | 1.000 | -1.046 | 0.296 |
| **Length** | -0.004 | 0.003 | -0.009 | 0.001 | 0.996 | 0.991 | 1.001 | -1.570 | 0.116 |
| **Head circumference** | -0.005 | 0.003 | -0.010 | 0.001 | 0.995 | 0.990 | 1.001 | -1.643 | 0.100 |
| **Abdominal circumference** | 0.006 | 0.002 | 0.002 | 0.011 | 1.006 | 1.002 | 1.011 | 2.632 | 0.009 |
| **Gestational age (weeks)** | -0.008 | 0.004 | -0.015 | -0.001 | 0.992 | 0.985 | 0.999 | -2.136 | 3.27E-02 |

**Supplementary table 4: Coefficients of the logistic regression model for HSP70 against hydrocephalus among preterm deliveries adjusted for the confounders GA, age at blood sampling, and neonatal anthropometrics (birth weight, length, head circumference, abdominal circumference).**

| HSP70 - non-CNS inflammatory ctrls vs ctrls | **Estimate** | **Std. Error** | **Lower 95%CI** | **Upper 95%CI** | **Fold change** | **FC lower 95% CI** | **FC upper 95% CI** | **t value** | **Pr(>\|t\|)** |
| --- | --- | --- | --- | --- | --- | --- | --- | --- | --- |
| **(Intercept)** | 11.809 | 0.087 | 11.638 | 11.979 |  |  |  | 135.947 | 0.00E+00 |
| **non-CNS inflammatory ctrls** | 0.031 | 0.012 | 0.007 | 0.054 | 1.031 | 1.007 | 1.055 | 2.560 | 0.010 |
| **Age at collection (days)** | -0.008 | 0.004 | -0.015 | -0.001 | 0.992 | 0.985 | 0.999 | -2.183 | 0.029 |
| **Birth weight** | 0.000 | 0.000 | 0.000 | 0.000 | 1.000 | 1.000 | 1.000 | -3.022 | 0.003 |
| **Length** | -0.001 | 0.002 | -0.005 | 0.002 | 0.999 | 0.995 | 1.002 | -0.787 | 0.431 |
| **Head circumference** | -0.007 | 0.002 | -0.011 | -0.003 | 0.993 | 0.989 | 0.997 | -3.558 | 3.75E-04 |
| **Abdominal circumference** | 0.007 | 0.002 | 0.004 | 0.010 | 1.007 | 1.004 | 1.010 | 4.005 | 6.23E-05 |
| **Gestational age (weeks)** | -0.001 | 0.002 | -0.005 | 0.002 | 0.999 | 0.995 | 1.002 | -0.750 | 0.453 |

**Supplementary table 5: Coefficients of the logistic regression model for HSP70 against non-CNS inflammatory controls compared to the remaining controls adjusted for the confounders GA, age at blood sampling, and neonatal anthropometrics (birth weight, length, head circumference, abdominal circumference).**

| HSP70 - HC vs non-CNS inflammatory ctrls | **Estimate** | **Std. Error** | **Lower 95%CI** | **Upper 95%CI** | **Fold change** | **FC lower 95% CI** | **FC upper 95% CI** | **t value** | **Pr(>\|t\|)** |
| --- | --- | --- | --- | --- | --- | --- | --- | --- | --- |
| **(Intercept)** | 12.277 | 0.265 | 11.758 | 12.796 |  |  |  | 46.348 | 4.52E-240 |
| **Hydrocephalus diagnosed under 2 yrs** | 0.241 | 0.093 | 0.058 | 0.424 | 1.273 | 1.060 | 1.529 | 2.585 | 0.010 |
| **Age at collection (days)** | 0.001 | 0.012 | -0.023 | 0.025 | 1.001 | 0.977 | 1.026 | 0.097 | 0.923 |
| **Birth weight** | 0.000 | 0.000 | 0.000 | 0.000 | 1.000 | 1.000 | 1.000 | 0.898 | 0.370 |
| **Length** | -0.015 | 0.006 | -0.026 | -0.003 | 0.986 | 0.974 | 0.997 | -2.519 | 0.012 |
| **Head circumference** | -0.009 | 0.007 | -0.022 | 0.005 | 0.991 | 0.978 | 1.005 | -1.252 | 0.211 |
| **Abdominal circumference** | 0.007 | 0.005 | -0.003 | 0.018 | 1.007 | 0.997 | 1.018 | 1.402 | 0.161 |
| **Gestational age (weeks)** | -0.001 | 0.008 | -0.016 | 0.014 | 0.999 | 0.984 | 1.014 | -0.133 | 0.894 |

**Supplementary table 6: Coefficients of the logistic regression model for HSP70 against hydrocephalus compared to non-CNS inflammatory controls adjusted for the confounders GA, age at blood sampling, and neonatal anthropometrics (birth weight, length, head circumference, abdominal circumference).**

| **Biomarker** | **Wilcoxon p-value** | **Bonferroni adjusted p-value** |
| --- | --- | --- |
| **logCRP** | 0.811 | 1 |
| **logIL18** | 0.004 | 0.037 |
| **logMCP1** | 0.327 | 1 |
| **logHSP70** | 0.004 | 0.036 |
| **logSTNF_RI** | 0.618 | 1 |
| **logEGF** | 0.099 | 0.988 |
| **logVEGF** | 0.116 | 1 |
| **logBDNF** | 0.378 | 1 |
| **logNT3** | 0.485 | 1 |
| **logS100B** | 0.640 | 1 |

**Supplementary table 7: Unadjusted and adjusted p-values comparing biomarker levels in hydrocephalus and non-hydrocephalus individuals.** Individuals diagnosed with hydrocephalus (n= 25) before the age of 1 years and those without hydrocephalus (n=14577) were analyzed. Non-parametric Wilcoxon rank-sum tests were used for group comparisons and Bonferroni-correction for multiple testing was applied.

| HSP70 - HC 1 year vs ctrls | **Estimate** | **Std. Error** | **Lower 95%CI** | **Upper 95%CI** | **Fold change** | **FC lower 95% CI** | **FC upper 95% CI** | **t value** | **Pr(>\|t\|)** |
| --- | --- | --- | --- | --- | --- | --- | --- | --- | --- |
| **(Intercept)** | 11.878 | 0.083 | 11.714 | 12.041 |  |  |  | 142.305 | 0.00E+00 |
| **Hydrocephalus diagnosed under 1 yr** | 0.294 | 0.090 | 0.117 | 0.470 | 1.341 | 1.124 | 1.601 | 3.254 | 0.001 |
| **Age at collection (days)** | -0.008 | 0.004 | -0.015 | -0.001 | 0.992 | 0.985 | 0.999 | -2.126 | 0.034 |
| **Birth weight** | 0.000 | 0.000 | 0.000 | 0.000 | 1.000 | 1.000 | 1.000 | -2.770 | 0.006 |
| **Length** | -0.002 | 0.002 | -0.005 | 0.002 | 0.998 | 0.995 | 1.002 | -0.982 | 0.326 |
| **Head circumference** | -0.008 | 0.002 | -0.011 | -0.004 | 0.993 | 0.989 | 0.996 | -3.892 | 1.00E-04 |
| **Abdominal circumference** | 0.007 | 0.002 | 0.003 | 0.010 | 1.007 | 1.003 | 1.010 | 3.912 | 9.22E-05 |
| **Gestational age (weeks)** | -0.002 | 0.002 | -0.006 | 0.001 | 0.998 | 0.994 | 1.001 | -1.297 | 0.195 |

**Supplementary table 8: Coefficients of the logistic regression model for HSP70 against hydrocephalus diagnosed before the age of 1 years adjusted for the confounders GA, age at blood sampling, and neonatal anthropometrics (birth weight, length, head circumference, abdominal circumference).**

| **Biomarker** | **Wilcoxon p-value** | **Bonferroni adjusted p-value** |
| --- | --- | --- |
| **logCRP** | 0.475 | 1 |
| **logIL18** | 0.286 | 1 |
| **logMCP1** | 0.422 | 1 |
| **logHSP70** | 0.746 | 1 |
| **logSTNF_RI** | 0.291 | 1 |
| **logEGF** | 0.965 | 1 |
| **logVEGF** | 0.948 | 1 |
| **logBDNF** | 0.824 | 1 |
| **logNT3** | 0.601 | 1 |
| **logS100B** | 0.397 | 1 |

**Supplementary table 9: Unadjusted and adjusted p-values comparing biomarker levels in hydrocephalus individuals with and without intraventricular hemorrhage.** Individuals diagnosed with hydrocephalus before the age of 2 years without intraventricular hemorrhage (n= 20) and hydrocephalus with IVH (n=10) were analyzed. Non-parametric Wilcoxon rank-sum tests were used for group comparisons and Bonferroni-correction for multiple testing was applied.

| **Biomarker** | **Wilcoxon p-value** | **Bonferroni adjusted p-value** |
| --- | --- | --- |
| **logCRP** | 0.688 | 1 |
| **logIL18** | 0.652 | 1 |
| **logMCP1** | 0.264 | 1 |
| **logHSP70** | 0.273 | 1 |
| **logSTNF_RI** | 0.662 | 1 |
| **logEGF** | 0.862 | 1 |
| **logVEGF** | 0.988 | 1 |
| **logBDNF** | 0.483 | 1 |
| **logNT3** | 0.409 | 1 |
| **logS100B** | 0.364 | 1 |

**Supplementary table 10: Unadjusted and adjusted p-values comparing biomarker levels in individuals with intraventricular hemorrhage without and with hydrocephalus.** Individuals diagnosed with intraventricular hemorrhage without (n= 105) and with hydrocephalus diagnosed before the age of 2 years (n=10) were analyzed. Non-parametric Wilcoxon rank-sum tests were used for group comparisons and Bonferroni-correction for multiple testing was applied.

| **Biomarker** | **Wilcoxon p-value** | **Bonferroni adjusted p-value** |
| --- | --- | --- |
| **logCRP** | 0.869 | 1 |
| **logIL18** | 0.099 | 0.994 |
| **logMCP1** | 0.776 | 1 |
| **logHSP70** | 0.018 | 0.181 |
| **logSTNF_RI** | 0.571 | 1 |
| **logEGF** | 0.227 | 1 |
| **logVEGF** | 0.116 | 1 |
| **logBDNF** | 0.451 | 1 |
| **logNT3** | 0.294 | 1 |
| **logS100B** | 0.164 | 1 |

**Supplementary table 11: Unadjusted and adjusted p-values comparing biomarker levels in individuals with hydrocephalus not caused by intraventricular hemorrhage and those without hydrocephalus.** Individuals diagnosed with hydrocephalus not caused by intraventricular hemorrhage diagnosed before the age of 2 years (n= 20) and those without hydrocephalus (n=14472) were analyzed. Non-parametric Wilcoxon rank-sum tests were used for group comparisons and Bonferroni-correction for multiple testing was applied.
